# Prospective Analysis of the Effects of Maternal Immune Activation on Rat Cytokines during Pregnancy and Behavior of the Male Offspring Relevant to Schizophrenia

**DOI:** 10.1523/ENEURO.0249-18.2018

**Published:** 2018-08-29

**Authors:** Brittney R. Lins, Jessica L. Hurtubise, Andrew J. Roebuck, Wendie N. Marks, Nadine K. Zabder, Gavin A. Scott, Quentin Greba, Wojciech Dawicki, Xiaobei Zhang, Christopher D. Rudulier, John R. Gordon, John G. Howland

**Affiliations:** 1 Department of Physiology, University of Saskatchewan, Saskatoon S7N 5E5, Saskatchewan, Canada; 2 Department of Medicine, University of Saskatchewan, Saskatoon S7N 5E5, Saskatchewan, Canada

**Keywords:** polyI:C, recognition memory, social behavior, reversal learning, touchscreen

## Abstract

Influenza during pregnancy is associated with the development of psychopathology in the offspring. We sought to determine whether maternal cytokines produced following administration of viral mimetic polyinosinic-polycytidylic acid (polyI:C) to pregnant rats were predictive of behavioral abnormalities in the adult offspring. Timed-pregnant Sprague Dawley rats received a single intravenous injection of 4-mg/kg polyI:C or saline on gestational day (GD)15. Blood was collected 3 h later for serum analysis of cytokine levels with ELISA. Male offspring were tested in a battery of behavioral tests during adulthood and behavior was correlated with maternal cytokine levels. Maternal serum levels of CXCL1 and interleukin (IL)-6, but not tumor necrosis factor (TNF)-α or CXCL2, were elevated in polyI:C-treated dams. PolyI:C-treated dams experienced post-treatment weight loss and polyI:C pups were smaller than controls at postnatal day (PND)1. Various behavior alterations were seen in the polyI:C-treated offspring. Male polyI:C offspring had enhanced MK-801-induced locomotion, and reduced sociability. PolyI:C offspring failed to display crossmodal and visual memory, and oddity preference was also impaired. Set-shifting, assessed with a lever-based operant conditioning task, was facilitated while touchscreen-based reversal learning was impaired. Correlations were found between maternal serum concentrations of CXCL1, acute maternal temperature and body weight changes, neonatal pup mass, and odd object discrimination and social behavior. Overall, while the offspring of polyI:C-treated rats displayed behavior abnormalities, maternal serum cytokines were not related to the long-term behavior changes in the offspring. Maternal sickness effects and neonatal pup size may be better indicators of later effects of maternal inflammation in the offspring.

## Significance Statement

Psychiatric pathology is complex, poorly understood, and often results in chronic illness. Many psychiatric conditions are believed to occur as a result of genetic and environmental factors. Gestational adversity such as inflammation in pregnancy may act as a priming experience for the later emergence of psychopathologies, and accurate identification of risk factors may advise early interventions. We sought to characterize long-term behavior effects in the offspring of rats exposed to an inflammatory event during pregnancy and relate these effects to the serum levels of relevant cytokines CXCL1, interleukin (IL)-6, and tumor necrosis factor (TNF)-α. Our results suggest that these maternal cytokines are not strongly related to offspring behavior outcomes, and other measures may have greater value as predictors of behavior outcomes.

## Introduction

Inflammation during pregnancy is associated with increased risk of various psychopathologies in the offspring (Brown et al., 2004a; Brown, 2006; Fineberg and Ellman, 2013). The association between inflammation and psychiatric illness was initially demonstrated through epidemiological studies where influenza outbreaks preceded an increase in schizophrenia spectrum disorders as cohorts that were *in utero* during the epidemic reached adulthood (Brown, 2012). Inflammation in pregnancy has since been linked to additional pathologies in the offspring including bipolar disorder and autism (Atladóttir et al., 2010, 2012; Parboosing et al., 2013; Jiang et al., 2016; Scola and Duong, 2017). Heterogeneity of the pathogens (viral, bacterial, parasitic) associated with psychiatric outcomes suggests the maternal immune response may mediate the effects on the developing offspring (Brown and Patterson, 2011). This hypothesis has been corroborated through prospective studies where maternal serum levels of interleukin (IL)-8/CXCL8, a cytokine with cellular attracting properties in the chemokine family, were elevated during the second trimester in pregnancies where the offspring developed a schizophrenia spectrum disorder (Brown et al., 2004b). Subsequent research implicated exposure to IL-8 *in utero* with abnormalities in the offspring’s brains including increased ventricular cerebrospinal fluid and decreased cortical volumes (Ellman et al., 2010). Increased tumor necrosis factor (TNF)-α in late pregnancy has also been linked to schizophrenia in the offspring (Buka et al., 2001). In addition, retrospective estimations of maternal IL-6 levels during pregnancy were predictive of performance in a working memory task and functional brain connectivity determined by magnetic resonance imaging (MRI) in two year old offspring (Rudolph et al., 2018). A second study that followed human pregnancies and offspring at 6 months of age found maternal inflammatory cytokines (IL-6, TNF-α, MCP-1) mediated an effect of maternal depressive symptoms on negative affect in the offspring (Gustafsson et al., 2018).

Systemic treatment of pregnant rodents or nonhuman primates with an immune stimulant such as the synthetic double-stranded RNA molecule polyinosinic-polycytidylic acid (polyI:C) induces various brain changes in the offspring reminiscent of psychiatric illness (Meyer et al., 2009, 2014; Piontkewitz et al., 2012). PolyI:C increases proinflammatory cytokines such as IL-1β, IL-6, CXCL1 (rodent homolog of IL-8), and TNF-α in maternal circulation (Meyer et al., 2006; Hsiao and Patterson, 2011; Ballendine et al., 2015). A mouse study showed a causal role for IL-6 in the development of the offspring’s psychopathology as administration of IL-6 alone resulted in abnormal offspring behavior and abnormalities could be prevented with concomitant administration of an IL-6 antibody (Smith et al., 2007). However, few studies have prospectively analyzed the relationship between increased maternal cytokines during pregnancy and behavior of the offspring. In one study with rats, dams that lost weight following polyI:C had significantly higher serum TNF-α than those that gained weight. Offspring of dams that lost weight had reduced sucrose preference but no significant changes in prepulse inhibition (PPI) or locomotor responses to amphetamine or MK-801 (Missault et al., 2014).

Improved understanding of the relationship between maternal serum cytokines and offspring phenotype has potential to impact psychiatric disease prevalence through screening and early intervention, yet there is a lack of prospective data correlating maternal inflammation with offspring behavioral phenotypes (Jiang et al., 2016). The present study assessed the effects of maternal polyI:C on acute cytokine elevations in the dams and subsequent behavioral abnormalities in the offspring. The cytokines analyzed were based on those previously indicated as relevant in the literature: IL-6, IL-8/CXCL1, and TNF-α as well as CXCL2 as a negative control (Brown et al., 2004b; Smith et al., 2007; Ellman et al., 2010; Missault et al., 2014; Ballendine et al., 2015; Scola and Duong, 2017). To assay behavior related to the positive symptoms of schizophrenia we used locomotor activity in response to a novel environment and the NMDA receptor (NMDAR) antagonist MK-801 (Zuckerman and Weiner, 2005; Howland et al., 2012; Giovanoli et al., 2013). For negative symptoms we used a spontaneous test sociability test (Bitanihirwe et al., 2010). Cognitive impairment was assessed using PPI of the acoustic startle response (Meyer et al., 2009; Howland et al., 2012; Ballendine et al., 2015), a crossmodal recognition memory battery (CMOR; Winters and Reid, 2010; Ballendine et al., 2015; Marks et al., 2016), a spontaneous oddity task (Bartko et al., 2007), and two operant conditioning procedures which assess visual discrimination, strategy set-shifting, and reversal learning (Zhang et al., 2012; Ballendine et al., 2015; Bryce and Howland, 2015). Our hypothesis was that maternal proinflammatory cytokines would correlate with behavioral deficits in the offspring with higher proinflammatory cytokine concentrations relating to a more severe behavior phenotype.

## Materials and Methods

### Animals

Timed-pregnant Sprague Dawley rat dams (*n* = 43) arrived at the animal holding facility on gestational day (GD)7. Dams were singly housed in standard polypropylene ventilated cages in a temperature controlled (21°C) colony room on a 12/12 h light/dark cycle (lights on at 7 A.M.) with food (Purina Rat Chow) and water available ad libitum. Following arrival, dams were left undisturbed until treatment on GD15. All procedures were conducted during the light phase and behavioral experiments were conducted on adult offspring of both sexes (*n* = 192 total, *n* = 121 males, and *n* = 71 females). Results from the female offspring will be presented in a subsequent paper. Experimental procedures were approved by the University of Saskatchewan Animal Research Ethics Board.

### Maternal treatments and blood samples

**Figure 1.**
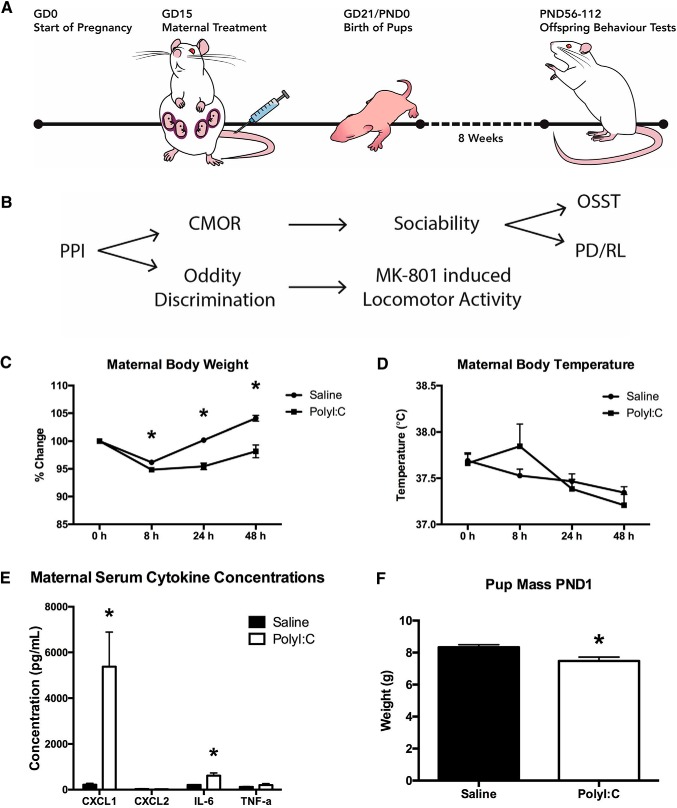
Acute effects of treatment on pregnant dams and neonatal offspring. ***A***, Schematic illustrating the timeline of maternal treatment and offspring behavior testing. ***B***, Schematic illustrating the division and order of offspring behavior testing. ***C***, Maternal weight loss was observed following the administration of polyI:C or saline. At all three time points measured (8, 24, 48 h), dams treated with polyI:C had a significantly lower percentage of their original body weight than those that received saline. ***D***, Maternal body temperature was slightly elevated in the polyI:C-treated dams 8 h following injection, yet this effect was not significant. ***E***, Maternal serum cytokine concentrations were determined using ELISA and revealed a significant increase in CXCL1 and IL-6 3 h after polyI:C treatment. No differences were seen for CXCL2 or TNF-α. ***F***, On PND1, the pups of dams treated with polyI:C weighed significantly less than those of the dams that received saline.

Maternal treatment generally followed previously established protocols in Long–Evans rats (Fig. 1*A*). On GD15, dam weight and rectal temperature were recorded. Dams were anesthetized with isoflurane (5% induction, 2.5% maintenance) for <10 min and received a single intravenous tail vein injection of either 0.9% saline or polyI:C (4 mg/kg, high molecular weight, InVivoGen). Dams were anesthetized a second time as described above 3 h following initial treatment and a blood sample (<1.5 ml total and <6% total blood volume) was drawn using a sterile catheter (BD Insyte Autogaurd, 24 GA 0.75 IN 0.7 × 19 mm, REF 381412) from the opposite tail vein used to inject polyI:C. Warm physiologic saline was administered once following polyI:C or saline treatment (3 ml), and a second time after blood collection (volume = the blood sample). Blood samples coagulated at room temperature for 1 h and spun at 10,000 × *g* for 5 min to separate the serum. Serum was pipetted and stored at -80°C until analysis and ELISAs were performed for CXCL1 (GROα/KC; R&D Systems), CXCL2 (GROβ/MIP-2; R&D Systems), IL-6 (PeproTech), and TNF-α (PeproTech) per the manufacturer’s instructions. Difficulty with blood collection of three polyI:C-treated dams resulted in small serum volumes collected for these rats and they were not included in IL-6 ELISA, and one was also excluded from TNF-α ELISA. While the offspring from these dams were included in behavioral testing, their behavior could not be correlated with these cytokine levels.

Aside from maternal weight and temperature monitoring 8, 24, and 48 h following treatment, dams were undisturbed for the remainder of their pregnancy. Of the original *n* = 43 dams, seven were euthanized within 48 h following polyI:C injection due to hypothermia. Four additional dams experienced body temperature below 36°C but otherwise showed alert behavior and were given access to a warming pad on their home cage for 24 h until their temperature returned to normal. Three dams did not produce viable litters. Ultimately, offspring from a total of *n* = 33 litters were included (*n* = 17 polyI:C-treated dams and *n* = 16 saline-treated dams). On postnatal day (PND)1, litters were weighed, sexed, and culled to a maximum of 10 (six males where possible). On PND23, pups were weaned and housed in same-sex sibling groups of two or three in standard housing as previously described with the addition of a plastic tube for enrichment.

### Behavioral testing

Behavioral tests were conducted according to published protocols or modified from published protocols. Typically, two males per litter were used in each behavior task. An exception is PPI, where three male offspring per litter were included, except for the two largest litters, where four and five were included. To account for the innate relationship between littermates, effects were averaged across siblings to produce one value per litter.

Rats were handled for a minimum of three sessions before behavior testing. Handling included exposure to investigators and emphasized picking up and moving the rats until these motions could be conducted with ease, as well as habituation to travel by cart between the animal housing and behavior testing locations. All work with the rats including husbandry and behavior testing occurred during the light phase (7 A.M. to 7 P.M.) with the majority of behavior testing performed between 8 A.M. and 5 P.M. Testing began at eight weeks of age (young adulthood) and was completed by 15 weeks of age. All offspring were first tested for PPI before being further divided into two groups for subsequent tests (Fig. 1*B*). One group (*n* = 68) completed CMOR and sociability before being assigned to complete either the operant set-shifting task (OSST, *n* = 37) or touchscreen pairwise discrimination and reversal learning (PD/RL, *n* = 38). The second group of randomly selected male rats (*n* = 30) from the same litters were tested in oddity discrimination followed by MK-801-induced locomotor activity. Ethanol (40%) was used to clean all behavior testing equipment between rats.

#### PPI

**Figure 2.**
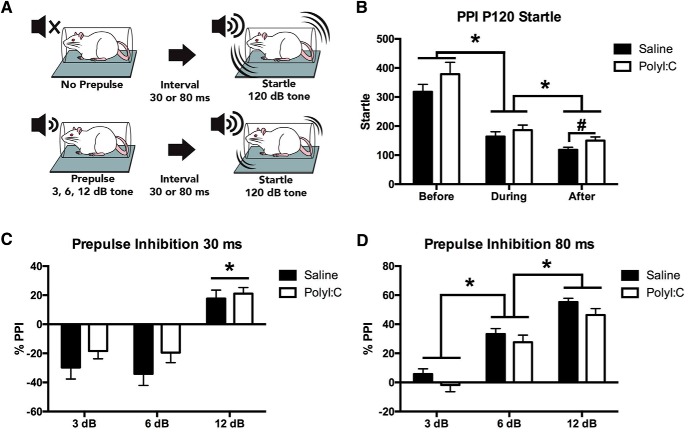
Effects of maternal polyI:C on acoustic startle and PPI in the offspring. ***A***, Illustration of the apparatus used to measure PPI. In the top panel no prepulse precedes the 120-dB tone and startle to the tone is high. The bottom panel highlights the typical reduction in startle response when the acoustic tone is preceded by prepulse of 3, 6, or 12 dB. ***B***, Offspring displayed a reduction in startle to the pulse alone trials with time where each time point was significantly different from the others as indicated with asterisks. Pairwise comparisons showed higher startle reactivity in the polyI:C offspring compared to controls at the “after” time point, indicated by a pound symbol (#). ***C***, No effect of treatment on PPI was seen for trials with a 30-ms prepulse-pulse interval. A main effect of prepulse intensity was seen with 12 dB producing greater PPI than 3 and 6 dB, indicated by asterisks. ***D***, No effect of treatment was seen for PPI on trials with an 80-ms prepulse-pulse interval. A main effect of prepulse intensity resulted in significantly greater PPI for trials with louder prepulses, indicated by asterisks.

PPI measures the percentage attenuation of motor response to a startling tone when that tone is preceded by a brief prepulse (Fig. 2*A*). Two SR-LAB startle boxes (San Diego Instruments) were used. Each session had a constant background noise (70 dB) and began with 5 min of acclimatization, followed by six pulse-alone trials (120 dB, 40 ms). Pulse-alone (6), prepulse + pulse (36), and no stimulus (6) trials were then presented in a pseudorandom order, followed by six additional pulse-alone trials. Prepulse + pulse trials began with a 20-ms prepulse of 3, 6, or 12 dB above background (70 dB). Prepulse-pulse intervals (time between the onset of the prepulse and the 120-dB pulse) were short (30 ms) or long (80 ms). The intertrial interval varied randomly from 3 to 14 s (Meyer et al., 2009; Howland et al., 2012; Ballendine et al., 2015).

#### Sociability task

**Figure 3.**
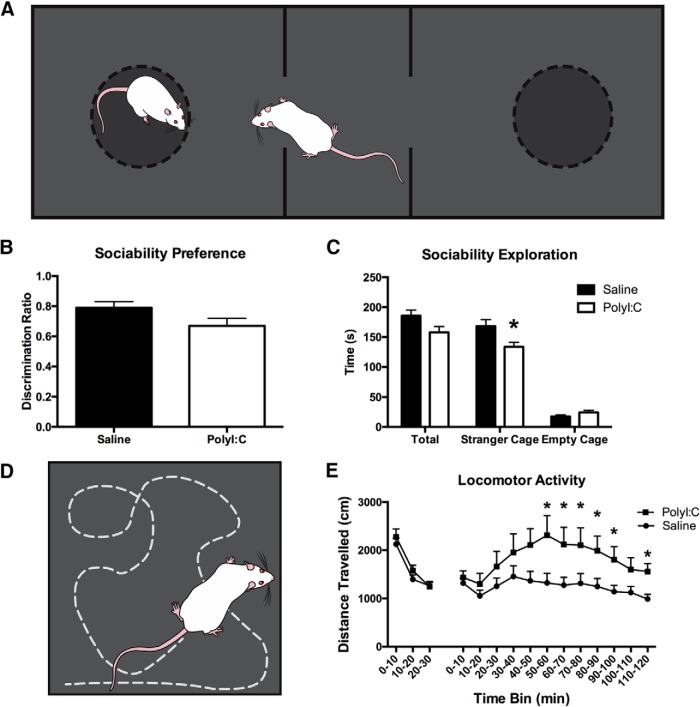
***A***, Schematic of the arena used for sociability testing. A black rectangular arena is divided into three sections where the two on the ends contain identical cages and are divided by a middle section. The stranger rat is held in one of the two cages and the test rat is free to explore the entire arena (for more detail, see Materials and Methods). ***B***, Social interaction behavior represented as a discrimination ratio. ***C***, When exploration time is divided into total exploration, stranger exploration, or empty cage exploration, polyI:C offspring spend significantly less time interacting with the stranger cage. ***D***, Schematic representing a rat in an open arena where locomotor activity was tracked over the course of a 30-min habituation and an additional 120 min following MK-801 injection. ***E***, PolyI:C offspring traveled a significantly greater distance than their saline counterparts following MK-801 treatment and the timepoints where a significant difference arose as determined by pairwise comparisons is indicated with an asterisk.

The testing apparatus was a rectangular arena (150 × 40 cm) of black corrugated plastic divided into three compartments, one middle start compartment (30 × 40 cm) and two “stranger” compartments on either side (60 × 40 cm; Fig. 3*A*). The walls dividing the middle compartment from the stranger compartments were clear Plexiglas (extended 12 cm from each wall leaving a 16-cm opening to move between compartments) and removable black opaque barriers which, when inserted, prevented entry into the stranger compartments. Each stranger compartment contained a circular mesh cage (18 cm in diameter, 20 cm in height) with hinged lid (3/4” plywood, painted matte black). The height of the cage was extended 20 cm with vertical metal rods to discourage climbing. The task began with 10-min habituation with the barriers removed. The test rat was then contained in the middle section with the barriers in place and a stranger rat was placed in one of the mesh cages. The barriers were removed, and the test rat explored for an additional 10 min. Interaction was scored when the face of the rat was oriented toward the holding cage at a maximum distance of 2 cm. Data were manually scored with a stopwatch by a blinded investigator and locomotor activity was recorded with EthoVision software. All stranger rats were sex, age, and treatment matched to the test rat (Henbid et al., 2017; Bitanihirwe et al., 2010).

#### Locomotor activity

The apparatus was a square arena (40 × 40 × 60 cm) made of black corrugated plastic (Fig. 3*D*). A camera mounted to the ceiling recorded all activity and EthoVision software was used to track activity. Rats were tested four at a time, with each rat placed in one of four separate arenas for 30 min of habituation. Immediately following, rats were administered MK-801 (0.2 mg/kg; i.p.; Howland et al., 2012) and placed back into the arena for an additional 120 min. Activity was recoded with Noldus Ethovision XT 11.5 software (Zuckerman and Weiner, 2005; Howland et al., 2012; Giovanoli et al., 2013).

**Figure 4.**
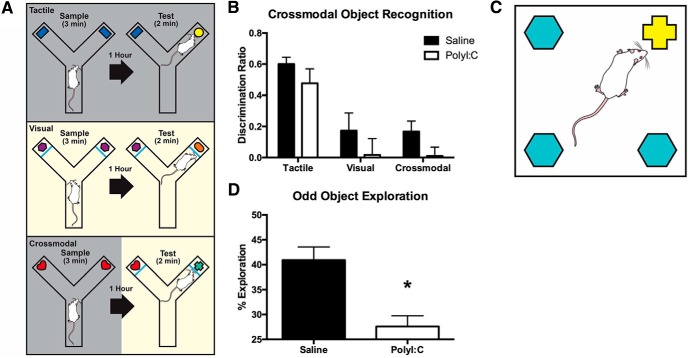
Maternal polyI:C treatment impaired multimodal, but not unimodal, object recognition memory in two spontaneous tasks. ***A***, Schematic of the crossmodal object recognition battery including the visual (top), tactile (middle), and crossmodal (bottom) variants. Each variant contains a sample phase with two identical objects and a test phase with one familiar object from the sample phase plus one novel object (for more detail, see Materials and Methods). ***B***, Offspring of both treatment groups display tactile memory as indicated by a positive discrimination ratio >0. Saline and polyI:C rats failed to perform above chance at visual memory while only saline rats displayed crossmodal memory. ***C***, Schematic of the oddity arena and object layout. A white square arena contained three identical objects and one odd object. Rats were allowed to explore the arena for 5 min following three habituation sessions (for more detail, see Materials and Methods). ***D***, Spontaneous oddity preference was significantly impaired in the polyI:C offspring.

#### Visual, tactile, and CMOR

This task uses spontaneous exploratory behavior to assess visual memory, tactile memory, and visual-tactile sensory integration (Winters and Reid, 2010; Jacklin et al., 2012; Marks et al. 2016). The testing apparatus was a Y-shaped maze with one start arm and two object arms (10 × 27 cm) made of white corrugated plastic (Fig. 4*A*). A white plastic guillotine-style door separated the start arm from the object arms, and Velcro at the distal end of the object arms fixed objects in place. A removable, clear Plexiglas barrier could be inserted in front of the objects. A tripod positioned above the apparatus held a video camera that recorded the task activity. Rats were habituated to the apparatus twice for 10 min. Lighting alternated during habituation between white light (used during visual phases) and red light (used during tactile phases) for 5 min each with the order counterbalanced, and the clear barriers were in place for one day of habituation and removed for the other with order counterbalanced between all rats. Test days consisted of a 3-min sample phase with two identical copies of an object attached with Velcro to the maze, a 60 min delay, and then a 2-min test phase with a third copy of the original object and a novel object placed in the maze. Rats began each phase in the start arm; the guillotine door was opened and closed once the rat entered the object arms. This task consisted of three distinct tests performed on three separate days; consistently in the following sequence: tactile memory (day 1), visual memory (day 2), and crossmodal memory (day 3). Red light illuminated the tactile phases allowing the rats’ behavior to be recorded while preventing the rats’ visual assessment of the objects and the removal of the clear barriers allowed for tactile exploration. White light was used during visual phases, but clear Plexiglas barriers in front of the objects prevented tactile exploration. CMOR had a tactile sample phase (red light, no barriers) and a visual test phase (white light, clear barriers). Recognition memory was defined as significantly greater exploration of the novel object than the familiar object. Behavior recordings were manually scored with a stopwatch by investigators blind to the treatment status of the rats and identity of the objects (Winters and Reid, 2010; Ballendine et al., 2015; Marks et al., 2016).

**Figure 5.**
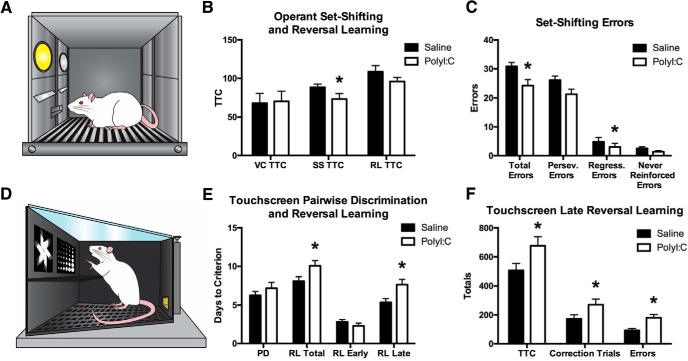
Performance of saline and polyI:C offspring on the operant task batteries PD/RL and OSST. ***A***, Illustration of the operant chamber with lights (visual stimuli) and levers used in OSST. ***B***, PolyI:C and saline offspring performed visual cue learning at the same rate in OSST. PolyI:C offspring were significantly facilitated at set-shifting indicated by the fewer TTC required. The subsequent reversal learning was unaffected by maternal treatment. ***C***, PolyI:C offspring made fewer perseverative errors and fewer regressive errors during set-shifting compared to control rats. ***D***, Illustration of the touchscreen chamber and stimuli used in the PD/RL task. ***E***, Bar graphs displaying the number of days required to complete each stage of the PD/RL task. Saline and polyI:C offspring learned visual paired discrimination at equal rates whereas touchscreen reversal learning (RL) required significantly more days of training for polyI:C rats to reach criterion. When divided into early and late RL, no differences were seen in the early stage while polyI:C rats were significantly impaired in the late stage. ***F***, Comparisons of the total number of trials completed, correction trials completed, and errors made during the late RL phase showed that the polyI:C rats were impaired on all of these measures.

#### Oddity discrimination

The testing apparatus was a square arena (60 × 60 × 60 cm) constructed of white corrugated plastic with Velcro in each of the four corners. Two days of habituation to the arena (10-min sessions) preceded the test day. On test day, three identical objects made of glass or plastic and one different, or “odd” object were fixed to the Velcro locations (Fig. 4*C*) and the rats’ activity were recorded for 5 min using a video camera mounted to the ceiling. Object exploration times were manually scored using a stopwatch by an investigator blind to the treatment status of the rats (Bartko et al., 2007). Object examination was counted when a rat’s face was oriented toward the object at a maximum distance of 2 cm.

#### OSST

Eight operant conditioning chambers (MedAssociates Systems) in sound-attenuating cubicles were used. The chambers contained two retractable levers and two stimulus lights positioned on either side of a food port (Fig. 5*A*) used to deliver food rewards (Dustless Precision Pellets, 45 mg, Rodent Purified Diet; BioServ). A 100-mA house light illuminated the chamber. Sessions began with levers retracted and the chamber in darkness (intertrial state), with the exception of lever training days during which the trial began with levers exposed to allow for baiting with ground reward pellets. Rats were tested once each day.

**Figure 6.**
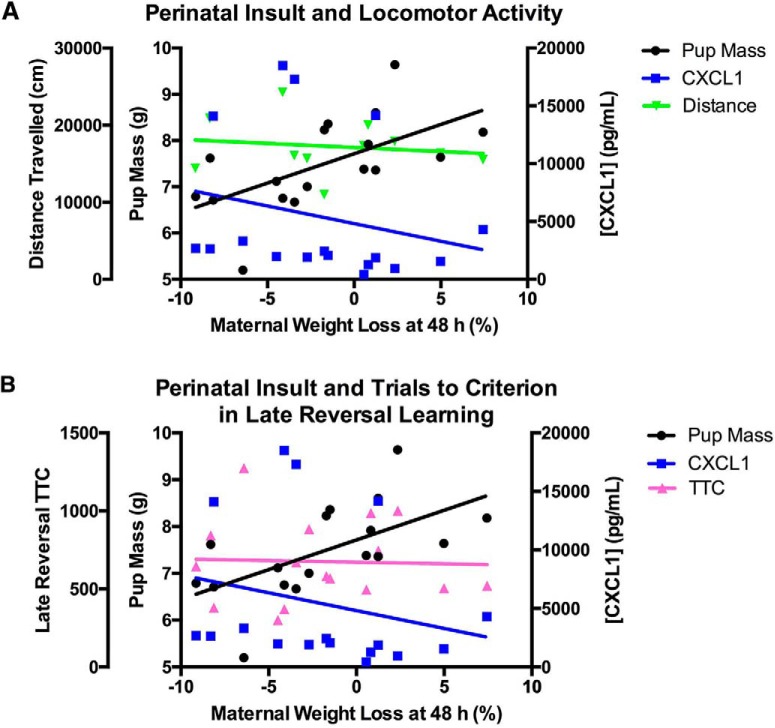
***A***, Overlaid regression plots showing the relationships between acute maternal weight loss at 48 h after polyI:C treatment and pup size, maternal CXCL1 concentration and distance traveled following MK-801 administration in polyI:C dams and offspring. Locomotor activity, used as an indicator of sensitivity to psychotomimetic compounds, was increased in the polyI:C offspring but no relationship to the measured indicators of inflammation or sickness in the pregnant dams was found. Weight change in the pregnant dams 48 h after polyI:C treatment was significantly correlated with pup size at birth (*r*_(17)_ = 0.60, *p* < 0.05). Serum CXCL1 was not related to weight change at 48 h (*r*_(17)_ = -0.23, *p* > 0.05). Distance traveled by the offspring in the locomotor task was not related to any of these (CXCL1: *r*_(13)_ = 0.34, *p* > 0.05; weight change 8 h: *r*_(13)_ = 0.30, *p* > 0.05; weight change 48 h: *r*_(13)_ = 0.23, *p* > 0.05; pup size: *r*_(13)_ = -0.07, *p* > 0.05; see also Table 2). ***B***, Overlaid regression plots showing the relationships between acute maternal weight loss at 48 h after polyI:C treatment and pup size, maternal CXCL1 concentration and trials to criterion in late reversal learning in polyI:C dams and offspring. Late reversal learning was chosen as a representative cognitive behavior test that was altered in the polyI:C offspring yet was not related to the measured indicators of inflammation or sickness in the pregnant dams (CXCL1: *r*_(17)_ = -0.30, *p* > 0.05; weight change 8 h: *r*_(17)_ = 0.003, *p* > 0.05; weight change 48 h: *r*_(17)_ = -0.04, *p* > 0.05; pup size: *r*_(17)_ = -0.05, *p* > 0.05; see also Table 2).

##### Lever training

Rats were trained to press the levers as described previously and immediately after reaching criterion, side preference was determined.

##### Visual-cue discrimination

Rats were trained to press the lever indicated by a stimulus light illuminated above it. Trials began with an illumination of one stimulus light, followed 3 s later by the house light and insertion of both levers. A correct press of the lever underneath the illuminated stimulus light caused retraction of both levers and the delivery of a reward pellet. An incorrect press returned the chamber to the intertrial state with no reward.

##### Strategy set-shift

The visual-cue rule from the previous stage was reinforced with 20 trials where the rat was required to press the lever below the illuminated stimulus light. Subsequently, rats were required to change their response from the visual cue to a spatial cue (the lever opposite to their side preference, regardless of whether the stimulus light was illuminated) to receive a reward pellet.

##### Reversal learning

Rats were required to press the lever opposite to the one rewarded during set-shifting. Criterion was 10 consecutive correct responses for each testing day (Floresco et al., 2008; Zhang et al., 2012; Thai et al., 2013; Ballendine et al., 2015).

#### PD/RL

Eight touchscreen-equipped operant conditioning chambers (Bussey-Saksida Touch Systems, Lafayette Instrument Company) in sound attenuating cubicles were used. The chambers were trapezoidal in shape with the wider end consisting of a touchscreen monitor (30.5 × 24.1 × 8.25 cm; Fig. 5*D*). Opposite the monitor was a food port for the delivery of food rewards. A roll-out shelf above the chamber contained a reward magazine and an overhead camera which provided a live feed of activity via output to an external monitor. The touchscreen monitor was covered with a polycarbonate mask with two rectangular windows that prevented contact with locations on the touchscreen irrelevant to the task. A response shelf extended 7 cm from the screen below the touchscreen windows and prevented unintentional touchscreen access. All training and testing was conducted per the manufacturer’s instructions and used the ABET II software that accompanied the chambers.

##### Pretraining

All protocols closely followed those recommended by the manufacturer and are previously published (Bryce and Howland, 2015). Rats were habituated to the chamber on two consecutive days with five reward pellets in the food port and the house light illuminated for 30 min. Criterion was reached if all pellets were consumed within 30 min. On the first day of task training, one food pellet was delivered every 30 s, as signified by a tone and illumination of the food port. During this stage of training, one of the touchscreen windows was illuminated pseudorandomly such that the same window was not illuminated for more than two consecutive trials. If the rat touched the illuminated screen, three reward pellets were delivered. In all stages of training and testing, each trial was preceded by a 20-s intertrial interval that was initiated once the rat’s nose entered the illuminated food port. During the second stage of training, the rat was required to nose poke the illuminated touchscreen window to receive a reward. Following this stage, the rat was required to nose poke into the illuminated port to initiate the illumination of a touchscreen window. Again, a reward was delivered if the rat then nose-poked the illuminated window. Criterion for these three stages was 100 trials in 1 h. Like the previous stages of training, the final stage of pretraining required initiation of the trial and the touching of the illuminated window to receive a food reward. However, touching the unilluminated window resulted in a 5-s time out followed by the intertrial interval. Trials that were incorrectly completed were followed by correction trials, whereby the same window was repeatedly illuminated until the correct selection was made and a food reward was delivered. Criterion was 100 trials completed in 1 h with a minimum of 80% correct for two consecutive days. Once criterion was reached, training on the full version of the task began.

##### Pairwise discrimination

Pairwise discrimination involved presentation of two distinct black and white images, one in each window of the screen. Each image could be presented in either location. One image was always correct, with its selection resulting in a food reward, regardless of its location (S+) while the other was always incorrect (S-). A correct choice resulted in presentation of a tone and illumination of the food port. An incorrect choice resulted in a 5-s delay followed by a correction trial. Rats repeated visual discrimination training daily until they reached a criterion of 100 trials completed in 1 h with ≥85% correct for two consecutive days.

##### Reversal learning

Reversal learning occurred following successful completion of pairwise discrimination. The protocol for reversal learning was identical to pairwise discrimination except the previously unrewarded stimulus (S-) was now the correct choice (S+), and the previous correct choice was now unrewarded and punished with a 5-s time out. Criterion was reached when the rat completed 100 trials in 1 h with ≥85% correct for two consecutive days.

### Statistical analyses

All figures show group means plus the standard error of the mean. A between-subjects design was used, and analyses were conducted with independent samples *t* tests and ANOVAs using Statistical Package for the Social Science version 22 (IBM). Significant time by treatment interactions in locomotor activity and maternal body weight were further analyzed with a priori pairwise comparisons to compare saline and polyI:C groups at each ordinal time point. Outliers were defined as having a performance metric falling >2 SDs from the mean and these were removed from analysis on a case by case basis.

Sphericity violations were accounted for using the Greenhouse–Geisser adjustment and t-tests were adjusted when Levene's test was violated. The use of one- and two-tailed tests is specified for each task. As mentioned, there is evidence that siblings are less variable than unrelated rats (Zorrilla, 1997; Lazic, 2013; Giovanoli et al., 2013) so sibling effects were averaged to produce one value per litter. Bivariate correlations were used to analyze the relationships between maternal serum concentrations of inflammatory cytokines, other acute sickness measurements, and offspring behavioral task performance. The false discovery rate correction (Benjamini–Hochburg (B–H)) was applied to grouped families of multiple comparisons; *p* ≤ 0.05 was considered significant.

## Results

### Effects of polyI:C treatment on the pregnant dams and neonatal offspring

PolyI:C treatment significantly affected acute maternal body weight changes measured as % change from baseline immediately before treatment (Fig. 1*C*). A repeated measures ANOVA revealed significant effects of time (*F*_(1.51,46.90)_ = 56.17, *p* < 0.001), treatment (*F*_(1,31)_ = 30.52, *p* < 0.001) as well as a time by treatment interaction (*F*_(1.51,46.90)_ = 16.91, *p* < 0.001). The polyI:C-treated dams had reduced body weight compared to the saline dams at 8 h (*p* < 0.01), 24 h (*p* < 0.001) and 48 h (*p* < 0.001) after treatment. Body temperature was not significantly affected by treatment (*p* > 0.05) although a main effect of time was observed (*F*_(2.28,70.72)_ = 8.91, *p* < 0.001) and no time by treatment interaction (*p* > 0.05; Fig. 1*D*). CXCL1 (*t*_(16.04)_ = -3.41, *p* < 0.01) and IL-6 (*t*_(27)_ = -2.62, *p* < 0.05) were significantly elevated in the polyI:C-treated dams (Fig. 1*E*). Neither CXCL2 nor TNF-α were affected by maternal treatment (*p* > 0.05). On PND1, pups from polyI:C-treated dams weighed significantly less than the saline pups (*t*_(31)_ = 2.93, *p* < 0.01; Fig. 1*F*), but there was no difference in litter size before culling to a maximum of 10 (saline = 11.94 ± 0.76; polyI:C = 12.00 ± 0.81; *t*_(31)_ = 0.56, *p* > 0.05). Pups were weighed once per week until weaning and the pup size difference was not seen at any other date (PND1: saline = 8.34 ± 0.16 g, polyI:C = 7.48. ± 0.28 g; PND7: saline = 22.98 ± 0.45 g, polyI:C = 22.10 ± 0.38 g; PND14: saline = 41.98 ± 0.74 g, polyI:C = 41.23 ± 0.76 g; PND21: saline = 75.85 ± 1.30, polyI:C = 75.40 ± 1.08 g).

### Maternal polyI:C treatment failed to significantly affect startle or PPI

Startle responses to acoustic stimuli were assessed by measuring startle alone and PPI in saline (*n* = 17 litters) and polyI:C offspring (*n* = 17 litters). Startle to the 120-dB pulses alone decreased during the session (main effect of time: *F*_(1.14,36.56)_ = 55.18, *p* < 0.001; Fig. 2*B*) but no treatment interaction was present. For prepulse trials with a 30-ms (short) interval, a main effect of prepulse intensity on PPI (*F*_(1.42,45.47)_ = 21.86, *p* < 0.001; Fig. 2*C*) was found with no effect of treatment. Overall, PPI was greater at 12 dB compared to 3 and 6 dB (*p* < 0.001). For trials with an 80-ms (long) prepulse-pulse interval, a main effect for prepulse intensity was found (*F*_(1.28,34.62)_ = 96.19, *p* < 0.001; Fig. 2*D*) for PPI, but no effect of treatment and no interaction. Overall, PPI increased with louder prepulses.

### PolyI:C offspring have reduced sociability

All offspring (*n* = 16 saline litters, *n* = 16 polyI:C litters) displayed a significant preference for the stranger cage (Fig. 3*B*; statistics not shown). PolyI:C offspring displayed a sociability deficit with less time exploring the stranger cage (*t*_(30)_ = 2.25, *p* < 0.05; Fig. 3*C*). There was no significant difference in total (stranger plus empty cage) exploration (*t*_(30)_ = 2.03, *p* > 0.05; Fig. 3*C*).

### Male polyI:C-treated offspring demonstrate heightened sensitivity to MK-801

Locomotor data obtained from polyI:C (*n* = 13 litters) and saline (*n* = 17 litters) offspring were analyzed with repeated measures ANOVAs. Results revealed a main effect of time (*F*_(2.73,76.54)_ = 7.67, *p* < 0.001; Fig. 3*E*). While the main effect of treatment failed to reach significance (*F*_(1,28)_ = 4.14, *p* = 0.051), a significant time by treatment interaction was observed (*F*_(2.73,76.54)_ = 2.87, *p* < 0.05). Differences between the groups arose following MK-801 injections where polyI:C offspring traveled greater distances than the saline counterparts (*p* ≤ 0.05).

### PolyI:C offspring perform tactile object recognition memory but not crossmodal object recognition memory

All CMOR data are presented as a discrimination ratio (Exploration_Novel_ – Exploration_Familiar_/Exploration_Total_) for the first minute of the test phase (*n* = 16 saline litters, *n* = 17 polyI:C litters). Both groups demonstrated significant tactile object recognition memory (saline: *t*_(15)_ = 13.70, *p* < 0.001; polyI:C *t*_(16)_ = 5.15, *p* < 0.001; Fig. 4*B*). Neither group performed above chance exploration for visual memory (saline: *t*_(14)_ = 1.54, *p* > 0.05; polyI:C *t*_(13)_ = 0.16, *p* > 0.05). In the crossmodal phase, saline offspring show significant preference for the novel object (*t*_(14)_ = 2.50, *p* < 0.05) while polyI:C do not (*t*_(16)_ = 0.21, *p* > 0.05). There were no differences in total object exploration times between groups in the sample and test phases (Table 1, statistics not shown).

### PolyI:C-treated offspring are significantly impaired in oddity discrimination

When analyzed with a one-sample *t* test, saline (*n* = 15 litters) offspring explored the odd object at a greater than chance level (*t*_(14)_ = 6.05, *p* < 0.001) but polyI:C offspring (*n* = 15 litters) did not (*t*_(14)_ = 1.20, *p* > 0.05). When the groups were compared directly with an independent samples *t* test, saline offspring spent a significantly greater % exploration with the odd object compared to polyI:C offspring (*t*_(28)_ = 3.92, *p* < 0.001; Fig. 4*D*). PolyI:C offspring spent a greater amount of time in total exploration of all objects compared to saline (saline: 67.81 ± 4.78 s; polyI:C: 84.08 ± 5.46 s; *t*_(28)_ = -2.24, *p* < 0.05).

### PolyI:C offspring have contrasting alterations in behavioral flexibility tasks

Saline (*n* = 16 litters) and polyI:C (*n* = 16 litters) offspring acquired the visual cue stage of OSST at the same rate (saline: 65.58 ± 10.93, polyI:C: 70.00 ± 12.20, *p* > 0.05). The polyI:C offspring had significantly facilitated set shifting performance as indicated by fewer trials required to reach criterion (*t*_(30)_ = 2.73, *p* < 0.05; Fig. 5*B*) and fewer total errors (*t*_(30)_ = 3.03, *p* < 0.01; Fig. 5*C*). Error breakdown revealed notable differences in perseverative errors with polyI:C offspring making marginally fewer (*t*_(30)_ = 1.90, *p* = 0.07; Fig. 5*C*), as well as a significant reduction in regressive errors *t*_(30)_ = 2.04, *p* = 0.05, but no difference in never reinforced errors (*t*_(23.14)_ = 1.85, *p* > 0.05). There were no differences in reversal learning, or other parameters of the task, including the 20 reminder trials for visual cue discrimination included on the first day of set-shifting (additional statistics not shown).

In the touchscreen PD/RL task, both groups learned the visual pairwise discrimination rule at the same rate with no differences in the number of days to criterion (DTC), total number of trials to criterion, total number of correction trials completed, or total number of errors made (statistics not shown). During reversal learning the polyI:C rats (*n* = 17 litters) required significantly more DTC than the saline offspring (*n* = 16 litters; *t*_(31)_ = -2.19, *p* < 0.05; Fig. 5*E*). No differences were seen for other measures. Reversal learning was then divided into early reversal (ER), which included all sessions before each rat achieved 50% correct during a single session, and late reversal (LR), which included all sessions afterward (Bryce and Howland, 2015). While there were no differences in ER, the polyI:C offspring required more DTC than the saline offspring in LR (*t*_(31)_ = -2.68, *p* < 0.05; Fig. 5*E*), required more TTC (*t*_(31)_ = -2.14, *p* < 0.05; Fig. 5*F*), completed more correction trials (*t*_(31)_ = -2.04, *p* = 0.05), and made more errors (*t*_(31)_ = -2.38, *p* < 0.05) than the saline offspring.

### Correlations between maternal cytokines, indicators of maternal sickness, and offspring phenotype

Maternal serum levels of IL-6, TNF-alpha, and CXCL2 were not related to measures taken from either the dams or offspring (correlations not shown). Maternal serum concentrations of CXCL1 were related to acute weight changes in dams treated with polyI:C at 8 h after injection (*r*_(17)_ = -0.51, *p* < 0.05); however, this effect was not robust following correction for multiple comparisons (B–H *p* > 0.05; Table 2). No relationships were seen between CXCL1 levels and weight loss at 24 h (*r*_(17)_ = -0.22, *p* > 0.05) or 48 h (*r*_(17)_ = -0.23, *p* > 0.05; Fig. 6) after injection, indicating the greater weight loss in dams with the highest elevations of CXCL1. No weight-CXCL1 correlations were seen in the saline rats.

**Table 1.** Total duration of object exploration during the sample and test phases for each variation of the CMOR task (mean ± SEM) for the adult offspring of dams treated with saline-or polyI:C on GD15 of pregnancy

Treatment	Task phase	Tactile	Visual	Crossmodal
Saline	Sample	44.97 ± 3.42	6.29 ± 0.45	44.68 ± 2.73
	Test	21.40 ± 1.52	3.40 ± 0.33	3.65 ± 0.55
PolyI:C	Sample	46.29 ± 2.36	7.10 ± 0.60	41.91 ± 2.67
	Test	19.64 ± 1.42	3.05 ± 0.40	3.10 ± 0.27

No significant differences were seen in any groups during the sample or test phases.

**Table 2.** Summary of the notable correlations between maternal and offspring measures taken in the present study. Correlations were grouped into 4 families to apply the B-H adjustment for multiple comparisons

Grouping	Correlation	Treatment	Unadjusted *p* value	B–H adjusted *p* value
Maternal-maternal	CXCL1-dam weight 8 h	Saline	0.28	0.40
		PolyI:C	0.04*	0.19
	CXCL1-dam weight 24 h	Saline	0.063	0.19
		PolyI:C	0.40	0.40
	CXCL1-dam weight 48 h	Saline	0.30	0.40
		PolyI:C	0.38	0.40
Maternal-neonate	Dam temp 8 h-pup mass PND1	Saline	0.36	0.36
		PolyI:C	0.04*	0.07
	Dam weight loss 48 h-pup mass PND1	Saline	0.35	0.36
		PolyI:C	0.01*	0.04*
Neonate-behavior	Pup mass-% oddity preference	Saline	0.16	0.32
		PolyI:C	0.02*	0.08
	Pup mass-locomotor	PolyI:C	0.81	0.81
	Pup mass-TTC LR	PolyI:C	0.25	0.33
Maternal-behavior	Dam weight 8 h-locomotor	PolyI:C	0.32	0.64
	Dam weight 48 h-locomotor	PolyI:C	0.46	0.69
	Dam weight 8 h-TTC LR	PolyI:C	0.99	0.99
	Dam weight 8 h-TTC LR	PolyI:C	0.89	0.99
	CXCL1-locomotor	PolyI:C	0.26	0.64
	CXCL1-TTC LR	PolyI:C	0.25	0.64

The unadjusted *p* values (α = 0.05) are presented alongside the false discovery rate (Benjamini–Hochburg) corrected *p* values. Significance in each column is indicated with an asterisk (*). TTC RL = trials to criterion late reversal; locomotor = distance traveled following MK-801 administration in the locomotor activity task.

Other effects of treatment on the dams correlated with pup phenotype. Change in maternal body temperature at 8 h after treatment was related to neonatal pup mass in polyI:C-treated rats (*r*_(17)_ = 0.51, *p* < 0.05) indicating a decrease in dam body temperature was related to the delivery of smaller pups; however this effect was not robust following adjustment for multiple comparisons (B–H *p* > 0.05; Table 2). No relationship was seen in saline rats. Dam weight change 48 h after polyI:C treatment was positively correlated with pup size at birth (*r*_(17)_ = 0.60, *p* < 0.05; Fig. 6), indicating a relationship between greater sustained weight loss in the dams and the delivery of smaller pups which was robust to the multiple comparison adjustment (B–H *p* < 0.05; Table 2). No relationship was found in the saline group. Pup mass at birth was then negatively correlated with oddity discrimination performance (percentage odd object exploration) in the polyI:C offspring only (*r*_(15)_ = -0.59, *p* < 0.05), interestingly showing smaller pups at birth had greater preference for the odd object despite reduced oddity preference as a group, although this effect is not seen following adjustment for multiple comparisons (B–H *p* > 0.05; Table 2). Oddity preference was not correlated to pup size in saline offspring. Other behavior effects were examined for relationships to sickness effects and maternal serum cytokines but no other relationships were found (representative results presented in Fig. 6*A*; bivariate correlation data of distance traveled by male polyI:C offspring following MK-801 administration with CXCL1: *r*_(13)_ = 0.34, *p* > 0.05; dam weight change 8 h: *r*_(13)_ = 0.30, *p* > 0.05; dam weight change 48 h: *r*_(13)_ = 0.23, *p* > 0.05; pup size: *r*_(13)_ = -0.07, *p* > 0.05; and see Table 2 for B–H adjusted *p* values; Fig. 6*B*; offspring trials to criterion in late reversal learning from PD/RL task; CXCL1: *r*_(17)_ = -0.30, *p* > 0.05; dam weight change 8 h: *r*_(17)_ = 0.003, *p* > 0.05; dam weight change 48 h: *r*_(17)_ = -0.04, *p* > 0.05; pup size: *r*_(17)_ = -0.05, *p* > 0.05; and see Table 2 for B–H adjusted *p* values).

## Discussion

This study describes the acute effects of intravenous administration of polyI:C to pregnant rats and relationships between individual maternal serum cytokine concentrations and behavioral outcomes in the adult offspring. PolyI:C administration elevated maternal serum concentrations of CXCL1 and IL-6, and caused weight loss. We did not observe an effect of polyI:C treatment on maternal body temperature at 8, 24, or 48 h after treatment, in accordance with several studies (Howland et al., 2012; Sangha et al., 2014; Ballendine et al., 2015), but not others (Zhang et al., 2012). Pups of polyI:C-treated dams weighed less than controls on PND1. In young adulthood, offspring behaviors were assessed using a behavior battery related to symptoms of psychopathology. PolyI:C offspring demonstrated behavioral changes in most tasks and serum cytokine levels in the pregnant dams correlated with weight loss, and bivariate correlations link this with offspring birth weight, however these effects appear to lack strong relationships with long-term offspring behavior effects.

One potential limitation of our results is the use of timed pregnant rats shipped to our facility on GD7. The effects of shipment during pregnancy on the dams and unborn offspring are a valid concern (Stewart and Kolb, 1988; Ogawa et al., 2007; Kuwagata et al., 2009; Meyer et al., 2009; Moriyama et al., 2013); nonetheless, the behavioral effects in the present cohort are comparable to those from in-house bred rat offspring, particularly on MK-801-induced locomotor behavior in the males (Zuckerman and Weiner, 2005; Vorhees et al., 2012). Timed-pregnant dams have been used in numerous similar studies that examine the roles of adverse gestational conditions and long-term effects on the offspring and constitute valuable contributions to the literature (Lodge and Grace, 2001; Du and Grace, 2013, 2016; Van den Eynde et al., 2014)

### Effects of polyI:C on pregnant dams and neonatal offspring

The double-stranded polyI:C molecule is recognized by the innate immune system in a similar manner as dsRNA via toll-like receptor 3 (TLR3). Subsequent nuclear factor κB-dependent signaling is best known for the induction of interferons; however, elevated serum concentrations of other immune proteins including IL-6, CXCL1, TNF-α, and IL-1β have been reported (Alexopoulou et al., 2001; Ballendine et al., 2015). Our results corroborate previous research with significant elevations of IL-6 and CXCL1 in maternal serum 3 h after polyI:C injection (Ballendine et al., 2015). A discrepancy exists in the effects of polyI:C on TNF-α as previous research reports Long–Evans rats, as well as Wistar rats that lost weight after treatment, showed elevated serum TNF-α (Missault et al., 2014; Ballendine et al., 2015). However, the polyI:C-treated Sprague Dawley dams did not have significantly elevated TNF-α levels in the present cohort. Elevated TNF-α is a well-established consequence of systemic inflammation when induced by polyI:C or other means such as LPS (Patterson, 2009; Mattei et al., 2014; Missault et al., 2014; Ballendine et al., 2015), although this effect is likely timing-dependent. A previous study demonstrated elevated TNF-α 2 h after LPS treatment which returned to control levels by 4 h. Both 3- and 6-h time points have been used by other groups and show mixed results (Smith et al., 2007; Cui et al., 2009; Missault et al., 2014; Ballendine et al., 2015). Given these variable results and the necessity of using a single time point in the present study, we cannot be certain whether TNF-α levels reached significant elevation at any time point in the polyI:C-treated dams. Weight loss following polyI:C treatment is robust in rats (Wolff and Bilkey, 2010; Piontkewitz et al., 2011a; Howland et al., 2012; Zhang et al., 2012; Missault et al., 2014; Sangha et al., 2014; Ballendine et al., 2015; Vorhees et al., 2015), consistent with established rodent sickness behaviors (Cunningham et al., 2007; Palin et al., 2009). We found that higher CXCL1 levels may be related to increased weight loss 8 h after polyI:C treatment although a greater sample size is needed to reduce the risk of type 1 error.

Maternal polyI:C-treated dams delivered pups with significantly lower body weight on PND1 compared to controls. While previous research in Long–Evans rats did not show this effect (Howland et al., 2012; Ballendine et al., 2015), low birth weight in humans is a risk factor for the development of psychopathologies including schizophrenia, affective psychosis, autism spectrum disorders, attention deficit/hyperactivity disorders, and impulsivity with conduct disorders (Jones et al., 1998; Moilanen et al., 2010; Moore et al., 2012; Grissom et al., 2014; Laurens et al., 2015; Van Lieshout et al., 2015; Mathewson et al., 2017). Increased maternal IL-6 during pregnancy is associated with lower birth weights in humans (Atta et al., 2016), and our correlation data revealed a robust relationship between sustained maternal weight loss (48 h) and lower offspring birth weight in a rat model as well.

### PolyI:C-treated offspring have behavior abnormalities associated with psychopathology

PPI is commonly used to assess sensorimotor gating which is altered in many psychiatric conditions and can be disrupted through the administration of dopaminergic and glutamatergic agonists. Earlier studies that assessed PPI in models of MIA showed PPI disruptions (for review, see Meyer et al., 2009); however, more recent work has failed to reproduce this effect (Missault et al., 2014; Van den Eynde et al., 2014; Vorhees et al., 2015). In agreement with several recent papers, our data showed no appreciable effect of MIA on PPI in the offspring.

PolyI:C offspring showed heightened sensitivity to MK-801. This finding replicates previous studies which report hyperlocomotion following MK-801 (Zuckerman and Weiner, 2005; Howland et al., 2012; Giovanoli et al., 2013), although some other studies have observed hypolocomotion (Vorhees et al., 2012, 2015; Missault et al., 2014). As the administration of NMDAR antagonists increases striatal DA efflux, exaggerated locomotor activity following MK-801 may relate to the positive symptoms of the disorder and increased striatal DA in schizophrenia patients (Usun et al., 2013; Laruelle, 2014).

Abnormal social functioning is seen in many patients with brain disorders (Kennedy and Adolphs, 2012), and we demonstrated a sociability deficit in adult polyI:C-treated offspring. Illnesses with a neurodevelopmental component like autism and schizophrenia have social deficits as a central feature, which fits with our finding that an early developmental insult impacted this behavior domain. Normal social functioning is dependent on neurodevelopmental processes as shown by the particularly deleterious effects of early life insult (Lee and Green, 2016). Early life deletion of the NR1 subunit, but not postadolescent deletion in mice results in impaired social preference and early childhood prefrontal cortex (PFC) damage in humans is associated with impaired sociability (Anderson et al., 2000). The network of regions with known roles in social cognition and social behaviors is diffuse, and include the PFC as well as the temporal lobe and amygdala, involved in facial and emotional recognition respectively, both of which are known to be altered in autism and are believed to contribute to the characteristic social deficits (Kennedy and Adolphs, 2012). The developmental effects of maternal inflammation are broad, and the impairments seen in our study could be related to the impact of developmental immune insult in one or several of these areas. NMDAR hypofunction induced by pharmacologic, genetic, and optogenetic means in mice disrupts preference for a stranger conspecific, and there is prior evidence for NMDAR disruption in MIA offspring as well as structural changes in the brain regions required for typical social proficiency, particularly the PFC (Samuelsson et al., 2006; Piontkewitz et al., 2011b; Lee and Green, 2016).

### PolyI:C administration during pregnancy results in altered cognition in behavior tasks in the adult offspring

The CMOR task assesses multisensory integration and the ability to form complex, multimodal representations of stimuli (Cloke et al., 2015; Jacklin et al., 2016). Previous research showed rats are capable of tactile-visual crossmodal memory indicated by greater visual exploration of a novel object compared to an object previously experienced tactilely (Winters and Reid, 2010). The role of the perirhinal cortex (PRh) and posterior parietal cortex (PPC) in visual and tactile recognition memory respectively is well known (Zhou and Fuster, 1997; Buckley and Gaffan, 1998; Murray and Richmond, 2001; Buckley, 2005; Albasser et al., 2011), although lesion studies have shown bilateral ablation of the PFC [medial PFC (mPFC) and orbitofrontal cortex (OFC)] selectively disrupts CMOR while leaving the control visual-visual and tactile-tactile memory tasks intact. More precise lesions reveal the necessary role of the OFC in CMOR (Winters and Reid, 2010; Reid et al., 2014; Cloke et al., 2015). We have previously demonstrated polyI:C offspring show specific deficits in CMOR (Ballendine et al., 2015). The present study with Sprague Dawley rats found a deficit in both visual and crossmodal domains of the task, effects suggesting maternal inflammation altered function of the OFC and PRh.

Oddity discrimination is a recently developed task that may assess the function of the ventral visual stream (VVS) and temporal lobe memory system in a spontaneous exploration paradigm. The perceptual-mnemonic/feature-conjunction neural network model suggests the PRh is situated at the most distal end of the VVS where it supports complex representations of an object comprised of simpler visual components which are hierarchically maintained in the more caudal regions of the VVS (Murray and Bussey, 1999; Bussey and Saksida, 2005; Bussey et al., 2005; Bartko et al., 2007). Visual learning and memory was highlighted as one of seven cognitive domains impaired in schizophrenia (Young et al., 2009), and inactivation of the PRh impairs oddity discrimination in rodents (Bartko et al., 2007), while structural abnormalities in the temporal lobe are seen in patients with schizophrenia as well as animal models including MIA (Piontkewitz et al., 2012). The oddity discrimination task reveals that polyI:C-treated offspring are impaired at performing a non-mnemonic visual discrimination with no delay, suggesting altered function of the VVS which feeds into the temporal lobe memory system, including the PRh. We observed a possible relationship where smaller pups had greater oddity preference but this was not robust when controlled for multiple comparisons.

Cognitive flexibility enables updating of appropriate behavioral responses following changing environmental demands. Set-shifting and reversal learning are related, yet dissociable behaviors used to measure cognitive flexibility (Floresco et al., 2009). In rodents, the operant set-shifting and reversal learning task (OSST) has been heavily studied and assesses both of these measures following the learning of a simple rule. Set-shifting requires inhibition of the initial behavior response pattern and adoption of a new strategy (extradimensional shift; EDS), and subsequently reversal learning requires performing the opposite behavior within the same dimension as the previous stage (intradimensional shift; IDS). In the paradigm used for this study, rats learned to press a lever indicated by an illuminated light (visual cue), strategy set-shift to press either the left or right lever regardless of visual stimuli (spatial cue), and finally reverse their behavior to press the opposite lever (Floresco et al., 2008, 2009; Thai et al., 2013; Ballendine et al., 2015; Brady and Floresco, 2015). We used a similar procedure in touchscreen operant chambers where rats learn to select one of two visual stimuli on a screen (pairwise discrimination) followed by reversal which required selection of the opposite stimuli (Bryce and Howland, 2015). Set-shifting behavior is known to depend on the mPFC, while reversal is orbito-frontal dependent (Floresco et al., 2009).

We observed a facilitation of performance in the set-shifting portion of OSST, characterized by less perseveration, less regression, and more rapid acquisition of a novel behavior strategy with no significant effect on RL. These results are inconsistent with some prior studies (Zuckerman and Weiner, 2005; Savanthrapadian et al., 2013) and impaired strategy set-shifting has been reported in the offspring of polyI:C-treated Long–Evans rats (Zhang et al., 2012; Ballendine et al., 2015). Another previous study however, reports subchronic ketamine administration before OSST resulted in reduced perseveration during set-shifting; however, this was accompanied with impaired learning of the initial discrimination and impaired reversal learning with increased perseveration when the present study reports no effect on initial cue learning or RL in this task paradigm (Floresco et al., 2009). In further contrast, polyI:C offspring were impaired in late reversal in the touchscreen PD/RL task. The specificity of impairment to late reversal suggests the polyI:C-treated offspring have difficulty identifying the new rule once they have ceased perseverating during ER learning, and impaired reversal learning in polyI:C offspring has been previously reported in spatial memory dependent tasks (Savanthrapadian et al., 2013; but see Zuckerman and Weiner, 2005). The role of DA in cognitive flexibility and behavior shifting is complex, where previous work suggests PFC DA depletion can facilitate EDS, yet blockade of D1 and D2 in the mPFC is known to impair with a specific increase in perseveration (Roberts et al., 1994; Ragozzino, 2002b; Floresco et al., 2006). The complex role of dopamine in cognitive flexibility is seen where the administration of amphetamine in rats results in impairments in set shifting, or impaired reversal learning with no effect on set shifting when compared across several studies (Weiner and Feldon, 1986; Russig et al., 2003; Fletcher et al., 2005). Abnormalities in DA transmission have been reported in MIA models including increased dopamine turnover and increased D2 binding (Ozawa et al., 2006), which may account for the changes in cognitive flexibility observed.

### Maternal serum cytokine levels following polyI:C treatment are not strongly related to offspring behavior outcomes

Prospective human studies suggest elevated cytokines in the maternal serum during pregnancy contribute to an increased risk of developing schizophrenia (Brown et al., 2004a,b; Brown, 2006, 2012; Ellman et al., 2010). In one sample, human IL-8 (and not IL-6, TNF-α, or IL-1β) was elevated in the serum of pregnant women whose offspring went on to develop schizophrenia (Brown et al., 2004b) and correlated with brain changes in the offspring (Ellman et al., 2010). In rodent models of MIA, maternal IL-6 released in response to MIA is necessary for schizophrenia-like symptoms in the mouse model (Smith et al., 2007) and administration of IL-6 alone to pregnant rats is sufficient to cause behavioral changes in the offspring (Samuelsson et al., 2006). Ballendine et al. (2015) also used an antagonist (G31P) for CXCL1 receptors (CXCR1/R2) in an attempt to block the behavioral effects of polyI:C in Long–Evans rats with mixed results. We sought to determine the relationship between individual maternal serum cytokine levels following an inflammatory insult and offspring phenotype to better understand the mechanisms underlying the behavioral effects of MIA (Meyer, 2014). Cytokines play diverse roles in brain development and affect functions such as induction and renewal of neuroepithelial cells which form scaffolding for migrating neurons, and neuronal migration (for review, see Deverman and Patterson, 2009; Stolp, 2013). IL-6 influences fate switching and cell differentiation in development while IL-6- and CXCL1-related signaling influences brain development by regulating neurogenesis, maturation, and survival (Ip, 1998; Nakashima et al., 1999; Cho and Miller, 2002; Ragozzino, 2002a; Gregg and Weiss, 2005; Deverman and Patterson, 2009; Arrode-Brusés and Brusés, 2012; Garay et al., 2013; Stolp, 2013).

In our study, the offspring of rats that experienced an immune challenge in pregnancy displayed a variety of behavior abnormalities that are associated with psychiatric and neurologic disease, yet individual maternal serum cytokines measured acutely following treatment with polyI:C were not strongly related to these effects. The altered behaviors are known to depend largely on overlapping brain regions, including the mPFC, OFC, striatum, amygdala, PRh, and postparietal cortex. Positive symptoms are often attributed to hyperactivity of the mesolimbic dopamine system while hypoactivity of mesocortical dopamine is linked to negative and cognitive symptoms (Abi-Dargham and Moore, 2003; Laruelle et al., 2003; Winterer and Weinberger, 2004; Guillin et al., 2007; Jarskog et al., 2007; Meyer and Feldon, 2009). Mechanisms by which LPS- and polyI:C-induced inflammation during pregnancy may influence development have been explored, and inflammatory events can lead to increased tyrosine hydroxylase in the nucleus accumbens of offspring, elevated dopamine, and reduced dopamine receptors in the PFC (Bacopoulos and Bhatnagar, 1977; Borrell et al., 2002; Romero et al., 2007; Meyer et al., 2008a, 2008b; Meyer and Feldon, 2009). These dopamine-related changes may be associated with increased IL-6 in development (Okubo et al., 1997a,b, Meyer et al., 2008a,b; Meyer and Feldon, 2009). Cognitive impairment is also linked to NMDAR signaling (Moghaddam et al., 1997; Moghaddam and Adams, 1998; Vinson and Conn, 2012). IL-6 elevations in late gestation increases NR1 expression in the adult hippocampus, although polyI:C induced maternal inflammation produced the opposite effect in the offspring (Samuelsson et al., 2006). The effort to identify individual causal inflammatory mediators may be challenging due to the diffuse effects of systemic inflammation in pregnancy. Multiple inflammatory and anti-inflammatory signaling pathways are initiated by TLR3 stimulation and the effects on neurodevelopment may still not be fully appreciated. We found significant correlations between acute serum CXCL1 and acute maternal weight loss, although these failed to translate to the broad offspring behavior abnormalities observed. Thus, our results suggest measures other than levels of individual circulating maternal inflammatory cytokines may be more informative of long-term behavioral outcomes of the offspring. For example, future experiments including multivariate analyses of an array of maternal inflammatory markers may provide more valuable predictive information about offspring behavior.

## Synthesis

Reviewing Editor: Liisa Galea, University of British Columbia Decisions are customarily a result of the Reviewing Editor and the peer reviewers coming together and discussing their recommendations until a consensus is reached. When revisions are invited, a fact-based synthesis statement explaining their decision and outlining what is needed to prepare a revision will be listed below. The following reviewer(s) agreed to reveal their identity: Amanda Kentner, Staci Bilbo.

The purpose of the present study was to determine whether rodent maternal serum cytokine levels following a gestational insult with Poly IC could be predictive of later life outcomes of male and female offspring (body weights, battery of translationally relevant behavioral tests). In general the authors report that maternal serum cytokine levels at time of challenge may be a poor indicator of later life endpoints in offspring and that alternative biomarkers/predictors must be explored. In general the manuscript was very well written but our specific questions and comments can be found below.

1. A major concern is that the Sprague-Dawley rat dams were timed-pregnant. While the authors indicate this as a limitation in their discussion, the use of timed-pregnant dams make it difficult to dissociate effects of inflammation versus stress, or stress + inflammation. The authors argue that the effects in the present cohort are comparable to those of Vorhees et al., 2012 (particularly for male MK-801 locomotor behavior), but there are significant differences between the outcomes of the studies, primarily with the PPI data. Relatedly, it is surprising that there were not robust treatment effects on PPI as this is an effect consistently reported for both rats and mice exposed to Poly IC prenatally.

2. Another major concern is the differential testing and analyses of both sexes. It appears as though the females were tested on all but 3 of the behaviors but the males were split up into 3 groups- 1) a subset of males tested on CMOR and sociability; 2) second subset tested on the OSST and pairwise/reversal training and 3) another group of males received the MK801 and oddity discrimination? This will have repercussions for analyses, as the sexes cannot be directly compared if they did not have the same behavioral experience.

If the females were not tested in the set shifting and pairwise discrimination, reversal learning tests - why not? Given that the sexes are given all of the other testing perhaps it would better to either not include the tests that were done only in males or only include male data in one paper and save the female data for another?

Often the sex is analyzed separately but this is missing an opportunity to discuss the sex differences seen in the findings. Given that schizophrenia has some sex differences in manifestation (including cognitive disturbances) it makes sense to investigate these, although with the caveat written above. Given that some of the sex differences seen in schizophrenia are age dependent, and that the testing was done over a long period ( 8 weeks to 15 weeks of age), are some of the more pronounced sex differences in the paper seen early on for males as in schizophrenia? This type of consideration could be helpful in determining whether the model recapitulates some of the sex differences seen in patients.

The locomotor activity did show some striking sex differences with males but not females showing a decrease in activity with MK801 but these are not highlighted as the authors suggest because different doses are used. While the reasoning is understandable it makes for a confusing read as the reader knows that both sexes were included but are not analyzed together and then sex differences cannot be discussed. This could be one of the highlights of the paper.

Then for oddity discrimination the sexes are pooled but this is not necessary in an ANOVA so it is not clear why this was done? We suggest either including all of the analyse with both sexes or as suggested about removing one sex from this paper as both sexes were not given all the same tests. Page 14, line 301, Did the authors mean “so effects were averaged across each litter and sexes were combined in instances where [NO] effects of sex were observed (oddity discrimination), either way it is confusing to the reader.

3. Please specify whether the Visual, Tactile and CMOR tests were counterbalanced or performed in a specific sequence. Page 10 (line 194) lists Visual, Tactile, then CMOR while line 209 lists as Tactile, Visual, then CMOR. In this regard, it would be helpful if the timeline included the order of behavioral testing.

4 The authors should discuss the limitation that there were not significant treatment effects for body temperature in this particular cohort of dams challenged with Poly IC. Is this something that should be considered with respect to cytokine levels and later offspring behavior.

Methods:

Which TNF was measured? If the authors do not wish to specify please provide a rationale for this reporting convention.

At what time during the light cycle did the behavioral tests take place?

Was the sex ratio of the litters altered with maternal treatment ( the authors discus other characteristics on page 15) but not sex ratio.

The use of the warming pad for a few dams could impact the immune response to the poly IC. Was this controlled for in the pup results?

In the methods the authors indicate that a 0.2 mg/kg i.p. dose of MK-801 was used (page 9, lines 175-176). However, in the results, they indicate that females received a different dose than males (page 16, lines 345-347). Please specify the dose used for each sex in the methods. Were dose response curves utilized to determine appropriate doses for each sex?

Statistics

Some outcomes are missing for example, what was the main effect of sex for social interaction?

Discussion, line 656: please clarify that it is individual variability in maternal serum cytokines that is being correlated to offspring outcomes. This is not clear as it is currently written. The authors in fact may want to include a more multivariate analysis such as network analyses or principle component analysis to understand how the changes in maternal cytokines could have influence pup behavior rather than correlation one at a time?

Page 19, line 409 - a p = 0.08 should not be presented as though it is indicative of a significant pattern. This occurs in a few places, namely Figures 5 and 6 to indicate non-significant differences between saline and polyIC animals. Is the reporting to indicate that these comparisons are non-significant, or if the intention is to suggest that there is 'almost' a significant difference, which is an incorrect assumption without reporting effect sizes.

It is not clear if post hocs were used, or other corrections for multiple comparisons. With respect to the Bivariate correlations did the authors utilize the False Discovery Rate correction?

Minor

In the figure caption for Figure 5 it looks like [A] and [D and E] have been reversed from the order of presentation in the figure.

The authors may wish to cite the following article: https://www.sciencedirect.com/science/article/pii/S0889159118302277?via%3Dihub Gustafsson, Sullivan, Loftis BBI 2018

What program was used for the manual scoring of the sociability task? Was the investigator blinded?
